# Simultaneous In Situ Imaging of pH and Surface Roughening during the Progress of Crevice Corrosion of Stainless Steel

**DOI:** 10.3390/s22062246

**Published:** 2022-03-14

**Authors:** Ko-ichiro Miyamoto, Rinya Hiramitsu, Carl Frederik Werner, Tatsuo Yoshinobu

**Affiliations:** 1 Department of Electronic Engineering, Tohoku University, Sendai 980-8579, Japan; hiramitsu.h27.rinya@gmail.com (R.H.); tatsuo.yoshinobu.a1@tohoku.ac.jp (T.Y.); 2 Department of Electronics, Kyoto Institute of Technology, Kyoto 606-8585, Japan; werner@kit.ac.jp; 3 Department of Biomedical Engineering, Tohoku University, Sendai 980-8579, Japan

**Keywords:** chemical sensor, light-addressable potentiometric sensor, stainless steel, crevice corrosion, pH imaging

## Abstract

Stainless steel plays an important role in industry due to its anti-corrosion characteristic. It is known, however, that local corrosion can damage stainless steel under certain conditions. In this study, we developed a novel measurement system to observe crevice corrosion, which is a local corrosion that occurs inside a narrow gap. In addition to pH imaging inside the crevice, another imaging technique using an infrared light was combined to simultaneously visualize surface roughening of the test piece. According to experimental results, the lowering of local pH propagated inside the crevice, and after that, the surface roughening started and expanded due to propagation of corrosion. The real-time measurement of the pH distribution and the surface roughness can be a powerful tool to investigate the crevice corrosion.

## 1. Introduction

Stainless steel is an essential material in industries due to its superior anti-corrosion characteristic, which results from a passivation film of native oxide formed on the surface. The damage of the passivation film is immediately recovered by oxidization. Products made of stainless steel withstand corrosion, even in seawater. In an underwater environment, the passivation film is maintained by dissolved oxygen [1].

It is known, however, that local corrosion of stainless steel may occur under certain conditions. Crevice corrosion is an example of local corrosion, which occurs inside a narrow crevice with a gap on the order of microns. Inside a crevice, protons are produced by hydrolysis of eluted metal ions, and dissolved oxygen is consumed to recover the passivation film. Due to the narrow geometry, where diffusion from inside or outside of the crevice is restricted, the solution inside has a low pH and low oxygen. In addition, chloride ions are attracted to maintain charge neutrality, and it is known that these chloride ions attack the passivation film. The lowering of pH inside the crevice is accelerated by the interaction of the processes above. When the pH value becomes lower than the critical value, “depassivation pH”, the passivation film can no longer be maintained, and the elution of the metal is further accelerated. The surface is then rapidly damaged by propagation of corrosion. It should be noted that the environment inside the crevice is not uniform, in general, reflecting the non-uniform geometry of the crevice, including in terms of its width and shape [2,3,4,5,6].

Although crevice corrosion has been extensively studied, experimental methods to probe inside such a narrow crevice have been limited due to the geometry. To overcome this limitation, Kaji et al. formed a gap between a test piece and a transparent glass plate, whose surface was modified with ion-sensitive dyes, and the spatial distributions of pH and chloride ions were optically monitored through the glass plate [7,8]. However, the measurable pH range of the dye is not wide enough, and the color change is almost invisible when the surface of the test piece is colored due to corrosion. There is a strong demand for an experimental method to observe the pH distribution inside crevices during the course of corrosion.

**Figure 1:**
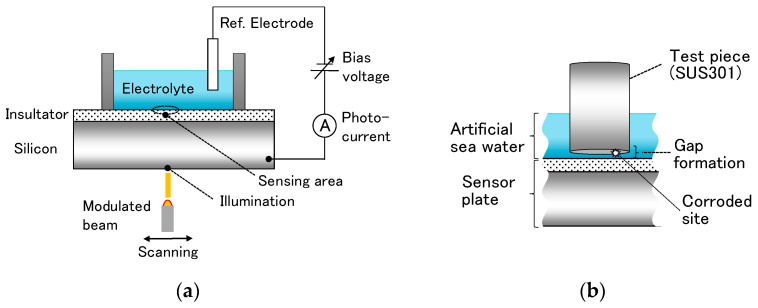
Schematic of the chemical imaging sensor system based on the LAPS principle: (**a**) the structure of the LAPS sensor plate and (**b**) the formation of a crevice with a narrow gap between the surface of the test piece and the sensor surface.

Recently, we proposed the label-free measurement of pH distribution inside a crevice [9,10,11] by applying a semiconductor-based chemical imaging sensor [12], which was based on the principle of the light-addressable potentiometric sensor (LAPS, [13,14]). Figure 1a shows a schematic view of a LAPS sensor plate. It has a flat sensor surface and a wide-range response to a pH change [9,10,11], which makes it an ideal sensor to monitor the pH change in the vicinity of the corroding surface of a test piece inside a crevice, as depicted in Figure 1b. Lowering of the local pH value was observed in the course of corrosion, which could be associated with the increase in the corrosion current. After the experiment, the corroded surface was optically inspected, and it was confirmed that the corroded area corresponded to the location where the lowering of pH was observed. However, the optical inspection was possible only ex situ, and it was not possible to identify the corroded area in the course of corrosion and to correlate it to the spatiotemporal change in pH distribution.

In this study, we developed a measurement system in which the pH distribution inside the crevice and the surface roughening of the test piece could be simultaneously visualized. Taking advantage of the fact that silicon is transparent to infrared light with a wavelength longer than ca. 1100 nm, additional optics was combined with the measurement system used in our previous study [10] to allow in situ inspection of the corroding surface of the test piece using an infrared light probe that penetrates the LAPS sensor plate.

## 2. Experiment

**Figure 2:**
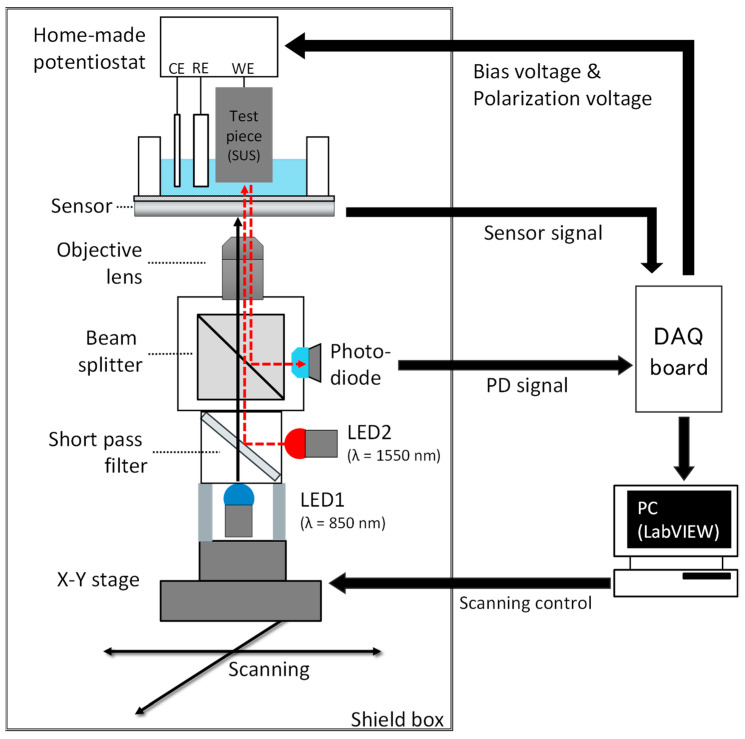
Measurement system.

Figure 2 shows the measurement system developed in this study. The system consists of (1) a measurement cell with its bottom made of a LAPS sensor plate; (2) a SUS304 test piece; (3) a home-made potentiostat; (4) scanning optics for probing pH and surface roughness; and (5) a control PC and measurement software. 

Sensor plate and measurement cell: The sensor plate was made of an *n*-type silicon substrate with double insulating layers of silicon dioxide (intermediate layer, 50 nm) and silicon nitride (pH-sensitive layer, 50 nm) on top. These insulating layers were formed by thermal oxidation and low-pressure chemical vapor deposition, respectively. The size and thickness of the sensor chip were 36 × 36 mm^2^ and 200 μm. The electrochemical cell was made of PVC, which was pressed onto the sensor surface with a rubber seal in between. The sensor plate was fixed on a metal plate that contacted the gold electrode evaporated on the back surface of the sensor plate. These are intrinsically the same as those used in our previous studies [9,10].

Test piece: A rod-shaped piece of JIS SUS304 (18–8 stainless steel) with a length of 25–35 mm and a diameter of 12 mm was used as a test piece. The composition of SUS304 used in this study was as follows: C 0.068%, Si 0.48%, Mn 1.84%, P 0.029%, S 0.027%, Ni 8.11%, Cr 18.65%. The surface of the test piece was first passivated by immersion in 35% nitric acid (FUJIFILM Wako Pure Chemicals Corp., Osaka, Japan) for 1 h at room temperature. The bottom surface was then polished with abrasive paper (200 mm in diameter, P600, KOVAX, Yokohama, Japan) directly before the experiment. The measurement cell was filled with artificial seawater (pH 7.8), in which the test piece was immersed and placed on the sensor surface to form a narrow crevice.

**Figure 3:**
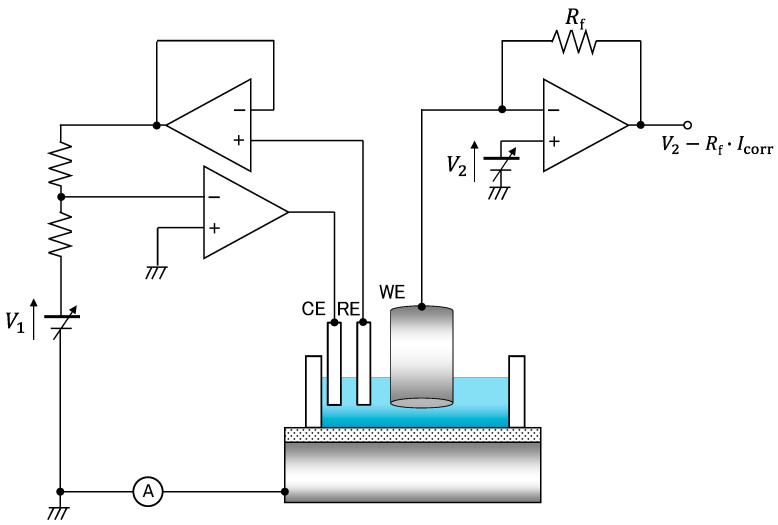
Circuit configuration of the homemade potentiostat.

Electrochemical system: The test piece is connected as a working electrode (WE) to the homemade potentiostat shown in Figure 3, together with an Ag/AgCl reference electrode (RE) and a platinum wire (CE). The LAPS sensor plate is virtually grounded via a transimpedance amplifier, and the potentials of the RE and the WE (test piece) with respect to the ground are set at *V*_1_ and *V*_2_, respectively. The circuit thereby polarizes the test piece at *V*_pol_ = *V*_2_ − *V*_1_ (vs. Ag/AgCl). The LAPS signal is obtained from the transimpedance amplifier, and the corrosion current *I*_corr_ is obtained by monitoring the output voltage, *V*_2_ − *R*_f_ *I*_corr_.

Scanning optics: As shown in Figure 2, two light beams are employed in the measurement system. A light beam from LED1 (L3989-01, Hamamatsu Photonics K.K.) has a wavelength of 850 nm, which excites the photocurrent signal in the LAPS sensor plate. The other light beam from LED2 (L12509-0155L, Hamamatsu Photonics K.K., Shizuoka, Japan) has a wavelength of 1550 nm, which penetrates the LAPS sensor plate and is reflected by the corroding surface of the test piece. The intensities of light beams from LED1 and LED2 are modulated at different frequencies of 2000 and 1500 Hz, respectively, so that they can be separated by lock-in detection to avoid cross-talk in the same manner described in [15].

The two light beams are mixed by a short pass filter with a cut-off wavelength of 1200 nm (Cat. #86-687, Edmund optics, Barrington, NJ, USA), which transmits and reflects the shorter and longer wavelengths, respectively. The two light beams are focused together by an objective lens (ULWD MIRPlan50, Olympus, Tokyo, Japan).

The light beam from LED2 specular reflected at the surface of the test piece penetrates the LAPS sensor plate again, and a part of it is guided to a photodiode (FCI-InGaAs-1000, OSI optoelectronics, Camarillo, CA, USA) by a beam splitter (#47-235, Edmund optics). The intensity of the specular reflected light beam, which decreases as a result of scattering by roughness or corrosion products, is measured by the photodiode as an indicator of the degree of local corrosion.

The optics is mounted on an X-Y stage to allow the light beams to two-dimensionally scan the LAPS sensor plate and the test piece. The photocurrent signal of the LAPS sensor plate and the current signal of the photodiode are converted into voltage signals by transimpedance amplifiers and recorded by PC via a data acquisition device (USB-6361, National Instruments, Austin, TX, USA). Maps of the pH distribution and corrosion are simultaneously obtained from these two signals.

## 3. Results and Discussion

**Figure 4:**
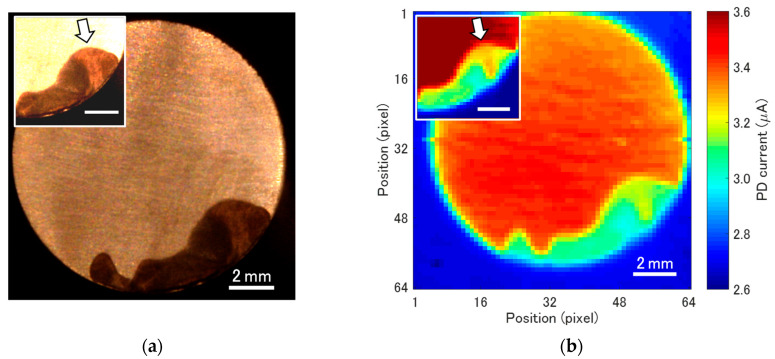
(**a**) An optical image of the corroded test piece and (**b**) the infrared reflection image observed through the LAPS sensor plate. Inset figures show the corroded area with enhanced contrast.

As a preliminary experiment, visualization of the corroded surface using the infrared light beam from LED2 was tested using a test piece which underwent crevice corrosion in advance. Figure 4a shows an optical image of the surface, in which a dark area shows the corroded area. Figure 4b shows a map of the intensity of the reflected light beam, which clearly correlates with the optical image in Figure 4a. In addition, the non-uniformity inside the corroded area in these two images match each other, as indicated by arrows in the insets. This result proves the possibility of infrared imaging of the surface through the LAPS sensor plate.

**Figure 5:**
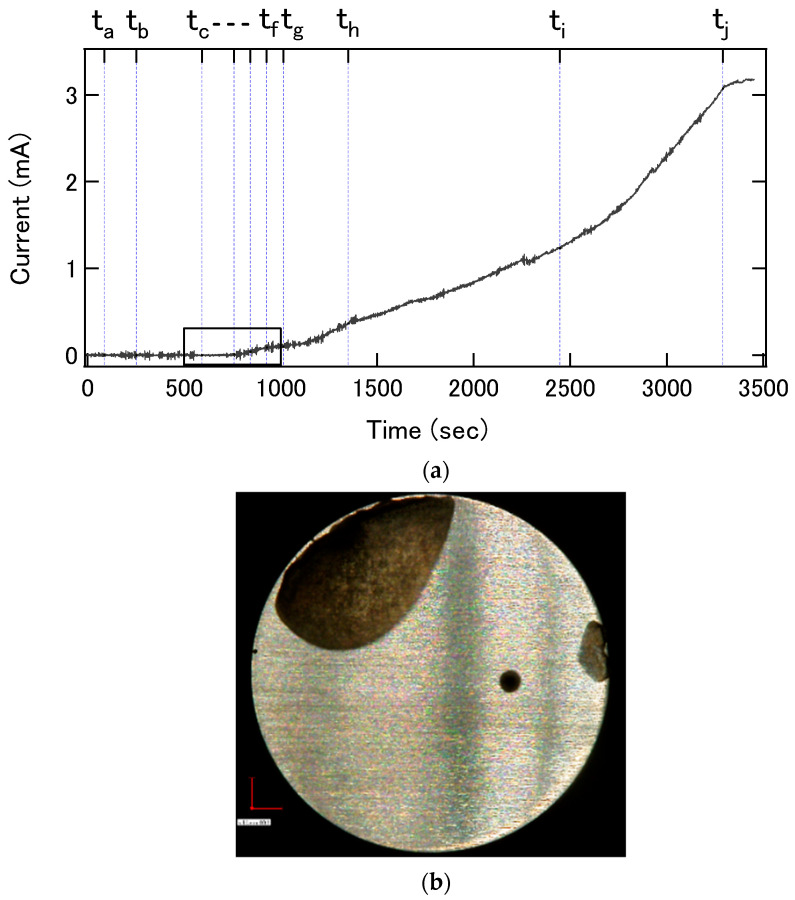
(**a**) The temporal change in the anodic current during the potentiostatic polarization of the test piece at 150 mV vs. Ag/AgCl in artificial seawater. The time stamps t_a_ to t_j_ correspond to the times at which the pH and roughness images in Figure 6a were recorded. A part of the curve surrounded by a rectangle, which corresponds to the initial stage of corrosion, is enlarged in Figure 7b. (**b**) Visual light image of the corroded surface after the experiment.

In situ measurement of crevice corrosion: The polished surface of a test piece was pressed against the sensor surface by its weight, leaving a narrow gap of approximately 12.3 μm [10]. The test piece was then potentiostatically polarized at 150 mV vs. Ag/AgCl in artificial seawater for 3400 s. The time course of the anodic current is shown in Figure 5a. The anodic current started to increase at around 760 s and continued to increase until the end of the experiment. Figure 5b shows the visible light image of the test piece after the experiment. Three areas on the surface were corroded; two were at the edge of the sample, and the other one was near the center of the sample.

**Figure 6:**
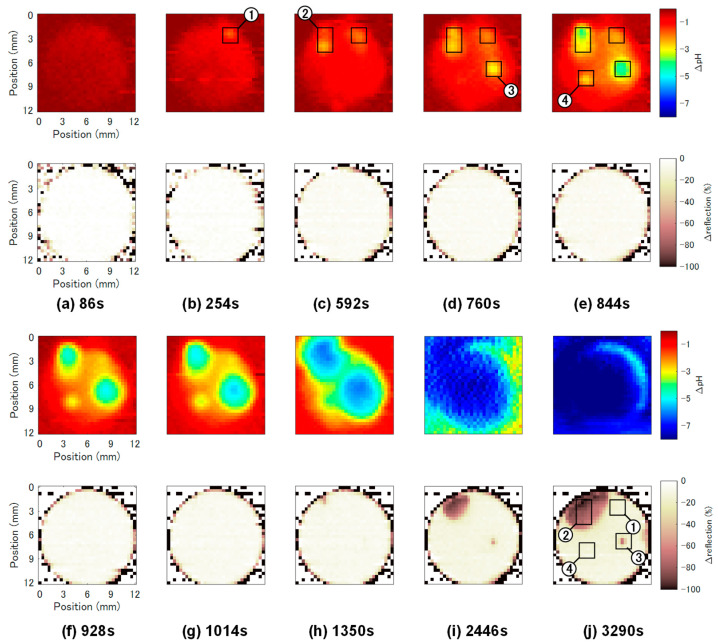
The change in pH distribution (**upper row**) and that of reflection (**lower row**). Areas 1 to 4 are the locations where the lowering of the local pH was observed.

During the potentiostatic polarization, two-dimensional distributions of pH and the intensity of reflected light, which indicates the roughness of the surface, were continuously acquired. The number of pixels, the scanning area, the sampling frequency, and the sampling number were 32 × 32, 12.8 mm × 12.8 mm, 100 kHz, and 2400, respectively. The pH and roughness images were recorded every 85 s. Images labelled with (a) to (j) in Figure 6 were collected at times indicated by the corresponding time stamps in Figure 5a. In this series of measurement, local pH changes were observed in four areas.

**Figure 7:**
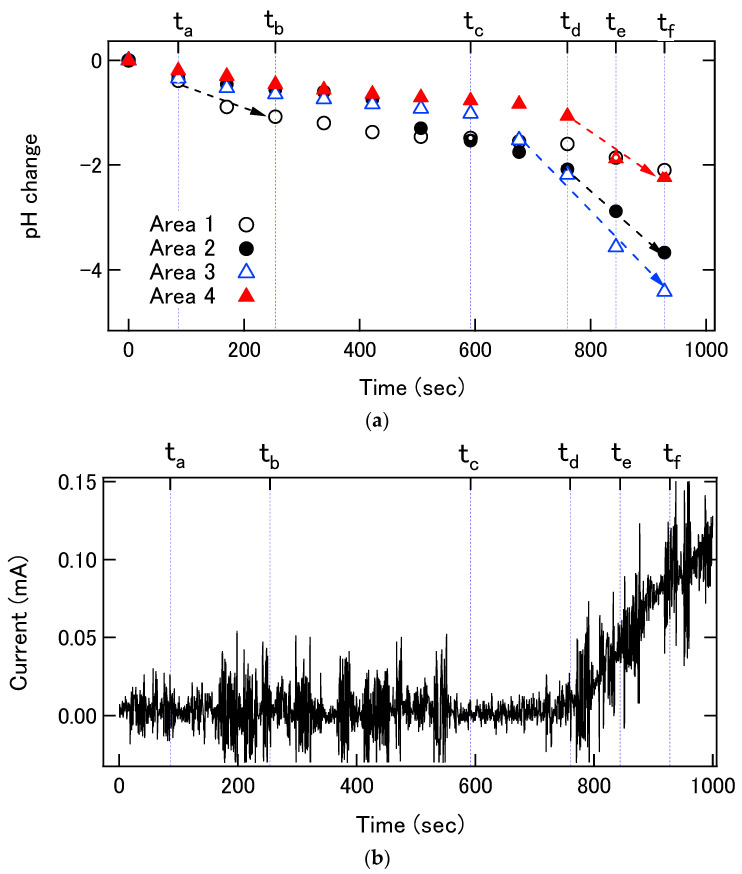
(**a**) pH changes in Areas 1 to 4 at the initial stage of corrosion. (**b**) Enlarged curve of the corrosion current shown in Figure 5a. (t_a_ = 86 s, t_b_ = 254 s, t_c_ = 592 s, t_d_ = 760 s, t_e_ = 844 s, t_f_ = 928 s.).

Figure 7a,b show the initial pH changes at Areas 1 to 4 and the increase in the corrosion current, respectively. The first obvious change in the local pH value was observed in Area 1 at 254 s (Figure 6b). At this stage, a major increase in the corrosion current was not yet detected in Figure 7b. Thereafter no further lowering of the local pH value was observed in Area 1, suggesting that this area was repassivated. Then, the local pH values started to lower successively in Area 2 at 592 s (Figure 6c) and Area 3 at 760 s (Figure 6d). In these two areas, the local pH values further continued to decrease, and the corroded areas expanded in the following pH images. The locations of these two areas correspond to the corroded areas observed in the optical image of the final surface shown in Figure 5b. Finally, the local pH value in Area 4 started to decrease at 844 s (Figure 6e), but the pH change in Area 4 was smaller than those in Areas 2 and 3. In Figure 7b, a major increase in the corrosion current was observed in synchronization with the acidification in Areas 2 and 3.

According to the mechanism of crevice corrosion, accelerated propagation of corrosion is triggered by elution of metal ions, which lowers the local pH value. Unless the elution is too much, the surface can be recovered by repassivation, consuming the remaining dissolved oxygen. The obtained results suggest the number of metal ions that were eluted at Areas 2 and 3 was greater than at Area 1. The position at which elution of metal ions occurs depends on the geometry of the crevice and the roughness of the sample surface. In addition, it is known that dissolution of nonmetallic inclusions at the surface can trigger pit corrosion, which will eventually initiate crevice corrosion. Manganese sulfide (MnS) is a typical inclusion in stainless steel [16,17,18]. Such inclusions included in the test piece may have determined the positions of pH reduction and corrosion.

**Figure 8:**
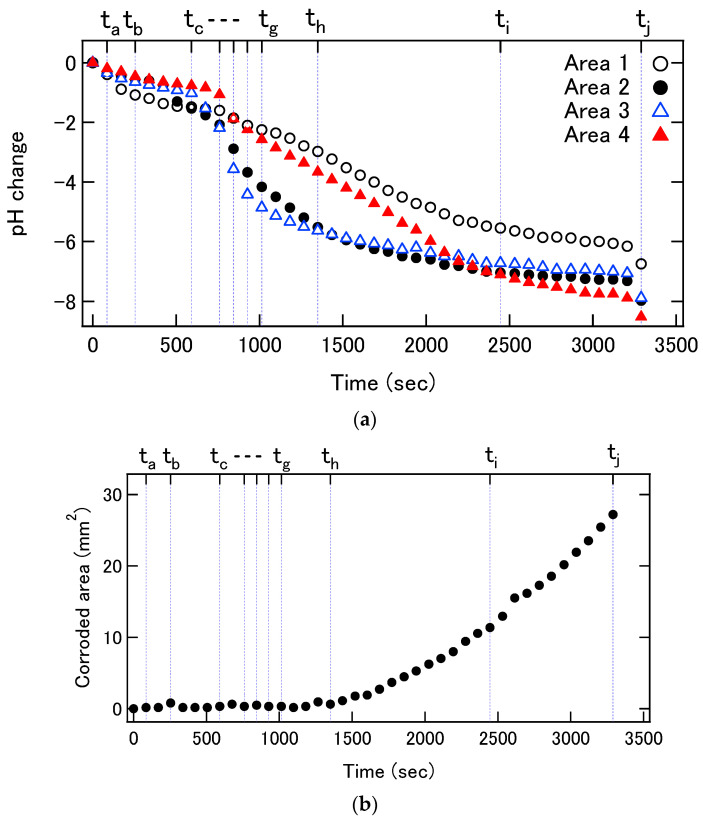

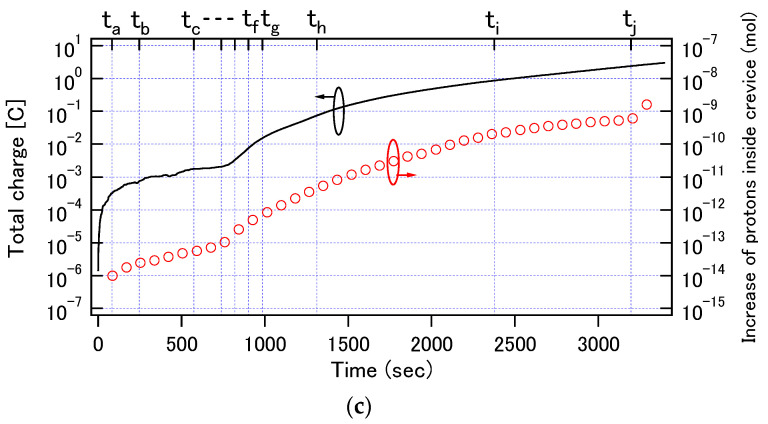
(**a**) Temporal changes in the averaged pH values in Areas 1 to 4. (**b**) Expansion of the corroded area detected by infrared reflection. (**c**) Time courses of the cumulative charge injected into the solution by the corrosion current and the increase in the number of protons inside the crevice calculated from the pH distribution.

Figure 8a shows the pH changes in these four areas for a longer period. The pH values in Areas 2 and 3 continued to decrease even after 1000 s, and eventually engulfed the minor pH changes in Areas 1 and 4, as observed in Figure 6.

According to the reflection images shown in Figure 6, the reflection in Area 2 decreased over 20% after 1350 s (Figure 6h), and then, the decrease propagated along the outer edge of the test piece. After 2446 s (Figure 6i), the reflection in Area 3 also decreased. After that, the reflection decreased at the right edge of the sample at 3290 s (Figure 6j). Figure 8b shows the temporal change in the total area of corrosion estimated from the reflection images by counting the pixels where the reflection decreased by more than 20%. Comparison of Figure 8a,b reveals that the onset of surface roughening at t_h_ is the time at which the local pH values at Areas 2 and 3 were approximately 2.2.

In order to illuminate the relationship between the corrosion current and the pH change, the following two quantities were calculated. The total increase in the number of protons inside the gap was calculated from the pH images by:

(1) $$\underset{\mathrm{all}\;\mathrm{pixels}}{\sum }\left\{\left(10^{-\mathrm{pH}\left(t\right)}-10^{-\mathrm{pH}\left(t=0\right)}\right)\times \mathrm{pixel}\;\mathrm{area}\;\times \mathrm{gap}\;\right\}\;\left[\mathrm{mol}\right],$$

where the pixel area was 16 × 10^4^ μm^2^ and the gap of the crevice was assumed to be 12.3 μm [7]. The total charge of ions eluted from the test piece was calculated from the corrosion current by:

(2) $$\overset{t}{\underset{0}{\int }}I_{\mathrm{corr}}\left(\tau \right)d\tau \;\left[C\right]$$

A proportional relationship was observed between the total charge injected by the corrosion current and the increase in the number of protons inside the crevice.

## 4. Conclusions

In this study, we developed a measurement system that is capable of visualizing both the pH distribution and the roughness inside a crevice. From the measurement results, the initial stage of crevice corrosion and its propagation were clearly observed. The lowering of the local pH value was synchronized to the increase in the corrosion current, and the surface roughening was observed after the local pH value was lowered. The combination of the chemical image sensor and the infrared light imaging through the sensor plate can be a powerful tool to study crevice corrosion.
